# Association of germline TYK2 variation with lung cancer and non‐Hodgkin lymphoma risk

**DOI:** 10.1002/ijc.34180

**Published:** 2022-07-09

**Authors:** James Yarmolinsky, Christopher I. Amos, Rayjean J. Hung, Victor Moreno, Kimberley Burrows, Karl Smith‐Byrne, Joshua R. Atkins, Paul Brennan, Colon Cancer Family Registry (CCFR), Colorectal Cancer Transdisciplinary Study (CORECT), Genetics and Epidemiology of Colorectal Cancer Consortium (GECCO), Prostate Cancer Association Group to Investigate Cancer Associated Alterations in the Genome (PRACTICAL) Consortium, James D. McKay, Richard M. Martin, George Davey Smith

**Affiliations:** ^1^ Medical Research Council Integrative Epidemiology Unit University of Bristol Bristol UK; ^2^ Population Health Sciences Bristol Medical School, University of Bristol Bristol UK; ^3^ Baylor College of Medicine Houston Texas USA; ^4^ Lunenfeld‐Tanenbaum Research Institute Sinai Health Toronto Ontario Canada; ^5^ University of Toronto Toronto Ontario Canada; ^6^ Biomarkers and Susceptibility Unit, Oncology Data Analytics Program Catalan Institute of Oncology (ICO), L'Hospitalet de Llobregat Barcelona Spain; ^7^ Colorectal Cancer Group, ONCOBELL Program Bellvitge Biomedical Research Institute (IDIBELL), L'Hospitalet de Llobregat Barcelona Spain; ^8^ Consortium for Biomedical Research in Epidemiology and Public Health (CIBERESP) Madrid Spain; ^9^ Department of Clinical Sciences, Faculty of Medicine University of Barcelona Barcelona Spain; ^10^ Cancer Epidemiology Unit, Oxford Population Health University of Oxford Oxford UK; ^11^ Genomic Epidemiology Branch, International Agency for Research on Cancer (IARC/WHO) Lyon France; ^12^ University Hospitals Bristol, NHS Foundation Trust, National Institute for Health Research Bristol Biomedical Research Centre, University of Bristol Bristol UK

**Keywords:** deucravacitinib, lung cancer, non‐Hodgkin lymphoma, tyrosine kinase 2

## Abstract

Deucravacitinib, a novel, selective inhibitor of TYK2 is currently under review at the FDA and EMA for treatment of moderate‐to‐severe plaque psoriasis. It is unclear whether recent safety concerns (ie, elevated rates of lung cancer and lymphoma) related to similar medications (ie, other JAK inhibitors) are shared with this novel TYK2 inhibitor. We used a partial loss‐of‐function variant in TYK2 (rs34536443), previously shown to protect against psoriasis and other autoimmune diseases, to evaluate the potential effect of therapeutic TYK2 inhibition on risk of lung cancer and non‐Hodgkin lymphoma. Summary genetic association data on lung cancer risk were obtained from a GWAS meta‐analysis of 29 266 cases and 56 450 controls in the Integrative Analysis of Lung Cancer Risk and Aetiology (INTEGRAL) consortium. Summary genetic association data on non‐Hodgkin lymphoma risk were obtained from a GWAS meta‐analysis of 8489 cases and 374 506 controls in the UK Biobank and InterLymph consortium. In the primary analysis, each copy of the minor allele of rs34536443, representing partial TYK2 inhibition, was associated with an increased risk of lung cancer (OR 1.15, 95% CI 1.09‐1.23, *P* = 2.29 × 10^−6^) and non‐Hodgkin lymphoma (OR 1.18, 95% CI 1.05‐1.33, *P* = 5.25 × 10^−3^). Our analyses using an established partial loss‐of‐function mutation to mimic TYK2 inhibition provide genetic evidence that therapeutic TYK2 inhibition may increase risk of lung cancer and non‐Hodgkin lymphoma. These findings, consistent with recent reports from postmarketing trials of similar JAK inhibitors, could have important implications for future safety assessment of deucravacitinib and other TYK2 inhibitors in development.

## Abstract 2

What's new?

Increased rates of lymphoma and lung cancer associated with Janus kinase (JAK) inhibitors used in the treatment of chronic inflammatory conditions have raised significant concern. A promising alternative, particularly for the treatment of plaque psoriasis, is deucravacitinib, a selective inhibitor of JAK family member TYK2. Here, the authors explored possible carcinogenic effects of TYK2 inhibition by genetic proxy based on a partial loss‐of‐function variant in TYK2 that provides protection against psoriasis. Analyses show that genetically proxied TYK2 inhibition increases lung cancer and non‐Hodgkin lymphoma risk. The findings could impact safety assessments of deucravacitinib and future novel TYK2 inhibitors.

**Abbreviations**
BCAC: Breast Cancer Association Consortium
CCFR: Colon Cancer Family Registry
CORECT: ColoRectal Cancer Transdisciplinary Study
FDA: Food and Drug Administration
GECCO: Genetics and Epidemiology of Colorectal Cancer Consortium
GWAS: genome‐wide association study
INTEGRAL: Integrative Analysis of Lung Cancer Risk and Aetiology
JAK: Janus kinase
LOF: loss‐of‐function
NHL: non‐Hodgkin lymphoma
PRACTICAL: Prostate Cancer Association Group to Investigate Cancer Associated Alterations in the Genome
TYK2: tyrosine kinase 2

## 1 INTRODUCTION

On September 1, 2021 the U.S. Food and Drug Administration (FDA) announced that three Janus kinase (JAK) inhibitors approved to treat chronic inflammatory conditions would require safety warnings over increased rates of serious heart‐related events, lung cancer and lymphoma.^1^ Recently, oral deucravacitinib, a selective inhibitor of tyrosine kinase 2 (TYK2, a member of the JAK family), was shown to lead to larger improvements in symptom severity for patients with moderate‐to‐severe plaque psoriasis than oral Apremilast, the current standard of care.^2^, ^3^ Deucravacitinib, therefore, has the potential to become an important treatment option for patients with psoriasis requiring systemic treatment and is currently under review for approval at the FDA and European Medicines Agency.^2^ It is unclear, however, whether the elevated cancer risk associated with some JAK inhibitors is shared with this novel TYK2 inhibitor.

In the absence of long‐term clinical trial data, naturally occurring genetic variation can be leveraged to validate therapeutic targets and predict their adverse effects.^4^ Specifically, germline mutations causing partial or complete loss‐of‐function (LOF) of genes encoding drug targets can be employed to mimic pharmacological inhibition of these targets and have been used to correctly predict adverse effects of new medications.^5^

Here, we used an established partial LOF mutation in *TYK2* (rs34536443), previously shown to protect against psoriasis and other autoimmune diseases,^6^, ^7^ to evaluate the potential effect of therapeutic TYK2 inhibition on risk of lung cancer and non‐Hodgkin lymphoma.

## 2 MATERIALS AND METHODS

Minor allele homozygosity of rs34536443 (observed in ~1 in 600 Europeans) causes near‐complete (~80%) loss of TYK2 function, while heterozygotes have a more modest reduction in function (<40%), suggesting nonadditive effects of this variant.^6^, ^8^, ^9^ To validate this variant as a surrogate for therapeutic TYK2 inhibition, we evaluated the effect of each copy of the minor allele of rs34536443, representing partial TYK2 inhibition, on risk of psoriasis, inflammatory bowel disease, Crohn's disease and multiple sclerosis. These analyses were performed using summary genetic association data on up to 78 334 cases and 150 030 controls from genome‐wide association studies (GWAS) of these autoimmune diseases.^10^, ^11^, ^12^, ^13^ Effect estimates were generated using the Wald ratio and standard errors were approximated using the delta method.^14^

To evaluate the effect of this variant on risk of overall and histological subtype‐specific lung cancer we obtained summary genetic association data on up to 29 266 cases and 56 450 controls from a GWAS meta‐analysis of the Integrative Analysis of Lung Cancer Risk and Aetiology (INTEGRAL).^15^ Summary genetic association data for non‐Hodgkin lymphoma (NHL) were generated by meta‐analysing genome‐wide association (GWAS) data for rs34536443 from the UK Biobank and InterLymph consortium in METAL.^16^

In secondary analyses, we explored whether rs34536443 increased risk of three other common adult cancers (breast, colorectal, prostate), which, along with lung cancer, account for approximately half of all new US cancer cases.^17^ Summary genetic association data on overall and histological subtype‐specific breast, colorectal and prostate cancer on up to 260 346 cases and 234 774 controls were obtained from analyses of the Breast Cancer Association Consortium (BCAC), Genetics and Epidemiology of Colorectal Cancer Consortium (GECCO), ColoRectal Cancer Transdisciplinary Study (CORECT), Colon Cancer Family Registry (CCFR) and the Prostate Cancer Association Group to Investigate Cancer Associated Alterations in the Genome (PRACTICAL) consortium.^18^, ^19^, ^20^

All analyses were restricted to participants of European ancestry. Further information on statistical analysis, imputation and quality control measures for these studies is provided in Appendix S1 and in the original publications.

## 3 RESULTS

To validate rs34536443 as a surrogate for therapeutic TYK2 inhibition, we confirmed that each copy of the minor allele, representing partial TYK2 inhibition, was associated with lower risk of psoriasis (OR 0.48, 95% CI 0.39‐0.58, *P* = 9.01 × 10^−14^) and other autoimmune diseases (Table 1).

**TABLE 1:** Effect of the minor allele of TYK2 variant rs34536443 on risk of autoimmune conditions, lung cancer and non‐Hodgkin lymphoma and other common adult cancers

Outcome	N (cases, controls)	OR (95% CI)	*P*‐value
*Autoimmune conditions*			
Psoriasis	10 588; 22 806	0.48 (0.39‐0.58)	9.01 × 10^−14^
Crohn's disease	5956; 14 927	0.65 (0.55‐0.76)	3.29 × 10^−7^
Multiple sclerosis	47 429; 68 374	0.77 (0.69‐0.85)	2.87 × 10^−7^
Rheumatoid arthritis	14 361; 43 923	0.68 (0.63‐0.75)	4.60 × 10^−16^
*Primary cancer outcomes*			
Lung cancer	29 266; 56 450	1.15 (1.09‐1.23)	2.29 × 10^−6^
Current or former smokers	23 223; 16 964	1.15 (1.07‐1.24)	1.72 × 10^−4^
Never smokers	2355; 7504	1.09 (0.91‐1.32)	.34
Lung adenocarcinoma	11 273; 55 483	1.17 (1.08‐1.27)	1.37 × 10^−4^
Squamous cell carcinoma	7426; 55 627	1.19 (1.08‐1.31)	2.98 × 10^−4^
Small cell lung cancer	2664; 21 444	1.16 (1.00‐1.34)	.05
Non‐Hodgkin lymphoma	8489; 374 506	1.18 (1.05‐1.33)	5.25 × 10^−3^
*Secondary cancer outcomes*			
Breast cancer	122 977; 105 974	0.99 (0.96‐1.03)	.69
ER+ breast cancer	69 501; 105 974	1.00 (0.96‐1.04)	.95
ER− breast cancer	21 468; 105 974	1.00 (0.96‐1.04)	.96
Colorectal cancer	58 221; 67 694	1.03 (0.99‐1.08)	.18
Colon cancer	32 002; 64 159	1.02 (0.97‐1.08)	.39
Rectal cancer	16 212; 64 159	1.05 (0.98‐1.13)	.15
Prostate cancer	79 148; 61 106	1.04 (1.00‐1.09)	.07
Advanced prostate cancer	15 167; 58 308	1.08 (1.00‐1.17)	.04

*Note*: OR represents the exponential change in odds of cancer per each copy of the minor allele of rs34536443. Advanced prostate cancer defined as Gleason score ≥8, prostate‐specific antigen >100 ng/mL, metastatic disease (M1) or death from prostate cancer.

In analyses of 29 266 lung cancer cases and 56 540 controls, each copy of the minor allele of rs34536443 was associated with an increased risk of lung cancer (OR 1.15, 95% CI 1.09‐1.23, *P* = 2.29 × 10^−6^). This association was stronger for current and former smokers (OR 1.15, 95% CI 1.07‐1.24, *P* = 1.72 × 10^−4^) compared to never smokers (OR 1.09, 95% CI 0.91‐1.32, *P* = .34). The magnitude of effect was similar across histology types. In analyses of 8489 cases and 374 506 controls, each copy of the minor allele of rs34536443 was associated with an increased risk of non‐Hodgkin lymphoma (OR 1.18, 95% CI 1.05‐1.33, *P* = 5.25 × 10^−3^). In secondary analyses, rs34536443 was weakly associated with risk of advanced prostate cancer, but not associated with other cancers examined. Exploratory analyses examining the association of rs34536443 with smoking initiation found little evidence to support an association (OR of ever smoking regularly: 1.00, 95% CI 0.99‐1.02, *P* = .67; Appendix S1).

## 4 DISCUSSION

Limitations to this analysis include the restriction of the majority of our analyses to summary genetic association data which precluded comprehensive assessment of effect modification across non‐lung cancer analyses. Second, the primary objective of these analyses was to evaluate safety profiles of TYK2 inhibition and, therefore, we did not include follow‐up assessment of potential pathophysiological mechanisms underpinning an effect of TYK2 inhibition on cancer risk. Finally, we were unable to compare the effect of genetically‐proxied TYK2 inhibition on cancer risk to that of genetically‐proxied inhibition of other JAK targets which was outside of the scope of this analysis.

The efficacy of deucravactinib in treating plaque psoriasis is attributed to the selective inhibition of TYK2, a downstream mediator of proinflammatory signalling pathways critical to psoriasis.^3^ Using an established partial loss‐of‐function variant to mimic therapeutic TYK2 inhibition, we show that potential protection from autoimmunity mediated by TYK2 inhibition may be counteracted by an increased risk of lung cancer and non‐Hodgkin lymphoma. This finding, including the restriction of an increased lung cancer risk to current and former smokers, is consistent with recent reports of higher rates of these two cancers in users of the JAK inhibitor Tofacitinib in the ORAL Surveillance safety trial.^1^ Importantly, the per‐allele estimates presented in this analysis may underestimate the effect of therapeutic TYK2 inhibition on cancer given larger TYK2 reductions achieved by deucravacitinib at doses shown to confer therapeutic benefit (50%‐80% TYK2 inhibition) compared to the <40% expected per copy of the rs34536443 minor allele.^21^ Meta‐analyses of observational studies have found strong evidence that individuals with psoriasis have an increased risk of lymphoma (RR 1.40, 95% CI 1.24‐1.57, 4 studies, *I* ^2^ = 23.3%) and weak evidence of an increased risk of lung cancer (RR 1.28, 95% CI 0.98‐1.68, 6 studies, *I* ^2^ = 87.7%).^22^ It is unclear whether this elevated risk reflects potential carcinogenic effects of chronic systemic inflammation seen in individuals with psoriasis, the use of immunomodulatory agents or the increased prevalence of known cancer risk factors (eg, smoking, excessive alcohol consumption and obesity) in individuals with psoriasis.^23^ Findings from our analysis therefore suggest that therapeutic TYK2 inhibition may increase risk of lung cancer and non‐Hodgkin lymphoma among populations who may already be at elevated risk of these diseases. Future exploration of mechanisms underpinning a putative effect of TYK2 inhibition in lung carcinogenesis could include genetic epidemiological assessment of the role of immune cell‐mediated cytokine signalling pathways previously shown to be impaired in TYK2‐immunodeficient patients in lung cancer risk (eg, type I interferon, IL10, IL12 and IL23 signalling).^8^ Our findings, suggesting potential adverse target‐mediated effects of TYK2 inhibition on lung cancer and non‐Hodgkin lymphoma, could have important implications for future safety assessment of deucravacitinib and other TYK2 inhibitors in development.

## AUTHOR CONTRIBUTIONS

*Conceptualization*: James Yarmolinsky, Richard M. Martin, George Davey Smith. *Formal analysis*: James Yarmolinsky, Kimberley Burrows, Karl Smith‐Byrne, Joshua R. Atkins. *Writing ‐ Original draft*: James Yarmolinsky. *Writing ‐ review & editing*: James Yarmolinsky, Christopher I. Amos, Rayjean J. Hung, Victor Moreno, Kimberley Burrows, Karl Smith‐Byrne, Joshua R. Atkins, Paul Brennan, James D. McKay, Richard M. Martin, George Davey Smith. The work reported in the article has been performed by the authors, unless clearly specified in the text.

## FUNDING INFORMATION

James Yarmolinsky is supported by a Cancer Research UK Population Research Postdoctoral Fellowship (C68933/A28534). James Yarmolinsky, Richard M. Martin and George Davey Smith are supported by a Cancer Research UK (C18281/A29019) programme grant (the Integrative Cancer Epidemiology Programme). James Yarmolinsky, Kimberley Burrows, Richard M. Martin and George Davey Smith are part of the Medical Research Council Integrative Epidemiology Unit at the University of Bristol which is supported by the Medical Research Council (MC_UU_00011/1, MC_UU_00011/5) and the University of Bristol. Richard M. Martin is also supported by the NIHR Bristol Biomedical Research Centre which is funded by the NIHR and is a partnership between University Hospitals Bristol NHS Foundation Trust and the University of Bristol. The US National Cancer Institute supports JM and PB (UO1CA203654) and JM (UO1CA257679). Department of Health and Social Care disclaimer: The views expressed are those of the author(s) and not necessarily those of the NHS, the NIHR, the International Agency for Research on Cancer or the Department of Health and Social Care. The funders had no role in the design of the study; the collection, analysis or interpretation of the data; the writing of the article; or the decision to submit the article for publication.

## CONFLICT OF INTEREST

Richard M. Martin was funded by a Cancer Research UK grant (C18281/A29019). The other authors declare no conflict of interest.

## ETHICS STATEMENT

Ethics and participant informed consent were obtained by each individual study contributing to GWAS; no separate ethics procedures were required for this analysis.

## Supporting information


**Appendix S1** Supporting Information.

## Data Availability

The data that support the findings of our study are available from the corresponding author upon reasonable request. Summary genetic association data for cancer endpoints were obtained from the INTEGRAL consortium (https://ilcco.iarc.fr/), PRACTICAL consortium (http://practical.icr.ac.uk/blog/) and GECCO consortium (https://www.fredhutch.org/en/research/divisions/public‐health‐sciences‐division/research/cancer‐prevention/genetics‐epidemiology‐colorectal‐cancer‐consortium‐gecco.html) via approved data usage proposals. Summary genetic association data on breast cancer risk can be downloaded from the Breast Cancer Association Consortium (https://bcac.ccge.medschl.cam.ac.uk/). Summary genetic association data on lymphoma from InterLymph were obtained via dbGaP accession number 15258. Summary genetic association data for the following autoimmune disease analyses were obtained from the GWAS Catalogue (https://www.ebi.ac.uk/gwas/): Crohn's Disease (Study accession: GCST003044), Rheumatoid arthritis (Study accession: GCST002318), Psoriasis (Study accession: GCST005527). Summary genetic association data on Multiple Sclerosis was obtained from the IEU GWAS Catalogue (https://gwas.mrcieu.ac.uk/datasets/ieu-b-18/).
